# Data-Sparse Prediction of High-Risk Schools for Lead Contamination in Drinking Water: Examples from Four U.S. States

**DOI:** 10.3390/ijerph20196895

**Published:** 2023-10-08

**Authors:** Samyukta Shrivatsa, Gabriel Lobo, Ashok Gadgil

**Affiliations:** Department of Civil and Environmental Engineering, University of California, Berkeley, CA 94720, USA; gplobo@berkeley.edu (G.L.); ajgadgil@berkeley.edu (A.G.)

**Keywords:** machine learning, lead, drinking water, environmental justice, open-source data mining

## Abstract

Childhood lead exposure through drinking water has long-term effects on cognition and development, and is a significant public health concern. The comprehensive lead testing of public schools entails high expense and time. In prior work, random forest modeling was used successfully to predict the likelihood of lead contamination in the drinking water from schools in the states of California and Massachusetts. In those studies, data from 70% of the schools was used to predict the probability of unsafe water lead levels (WLLs) in the remaining 30%. This study explores how the model predictions degrade, as the training dataset forms a progressively smaller proportion of schools. The size of the training set was varied from 80% to 10% of the total samples in four US states: California, Massachusetts, New York, and New Hampshire. The models were evaluated using the precision-recall area under curve (PR AUC) and area under the receiver operating characteristic curve (ROC AUC). While some states required as few as 10% of the schools to be included in the training set for an acceptable ROC AUC, all four states performed within an acceptable ROC AUC range when at least 50% of the schools were included. The results in New York and New Hampshire were consistent with the prior work that found the most significant predictor in the modeling to be the Euclidean distance to the closest school in the training set demonstrating unsafe WLLs. This study further supports the efficacy of predictive modeling in identifying the schools at a high risk of lead contamination in their drinking water supply, even when the survey data is incomplete on WLLs in all schools.

## 1. Introduction

Over half of the American population is estimated to have been exposed to adverse lead levels in early childhood [1]. Childhood lead exposure has been associated with several negative effects on a child’s IQ, attention span, learning ability, physical growth, and auditory capacity [2]. The lead from public water systems can constitute up to 80% of a child’s daily exposure to lead [3]. Hence, the Lead and Copper Rule Revisions (LCRR) that went into effect in 2021 emphasized the need for the comprehensive testing of drinking water sources for elevated water lead levels (WLLs) in elementary schools and child-care facilities [4].

LCRR advocates for a phased testing strategy where drinking water treatment plants test 20% of the schools and 20% of the licensed childcare facilities under their jurisdiction every year starting from 2025. However, the strategies for such an implementation have not been detailed and concerns have been raised regarding the feasibility of this venture. Given the high cost and time involved in implementing comprehensive school-wide lead testing programs, there is a clear need for a conceptual framework that guides the process, and prioritizes measurement at sites at risk of high WLLs.

The random forest modeling approach outlined in Lobo et al. 2022 [5] presented a novel framework that leveraged the existing data commonly available through different public agencies, thus saving both the time and expenditure involved in the collection of primary data. The study was based on publicly available WLL datasets published by the states of California and Massachusetts. A total of 70% of the sampled sites in each state were used to train the model, which achieved average ROC AUC and PR AUC scores of 0.88 and 0.91 (for Massachusetts) and 0.77 and 0.88 (for California). These modeling scores were significantly better than the prior efforts in the literature. Owing to the availability of complete datasets of WLLs in all schools in the states of California and Massachusetts, model predictions could be validated with real-world WLL measurements.

However, most of the remaining states do not have such comprehensive WLL datasets for all schools. Additionally, many states place the responsibility of testing on individual schools, but lack state-level resources and funding for testing [6]. For random forest modeling to be deployed in states with sparse data availability, a quantitative assessment of how predictions degrade with reduced training data for the model is necessary. This would allow agencies in states with limited lead testing data to develop a baseline for initial testing to ensure a reasonable predictive accuracy following the model deployment. As more lead data is obtained to train the model, model predictions can better direct future testing efforts to schools identified as being at a high risk for unsafe WLLs. Therefore, the objectives of this study were to assess (1) the degradation of the model predictions with a reduction in the training dataset sizes, and (2) how the relative importance of the predictors might change with decreasing the training set sizes.

## 2. Materials and Methods

**Figure 1:**
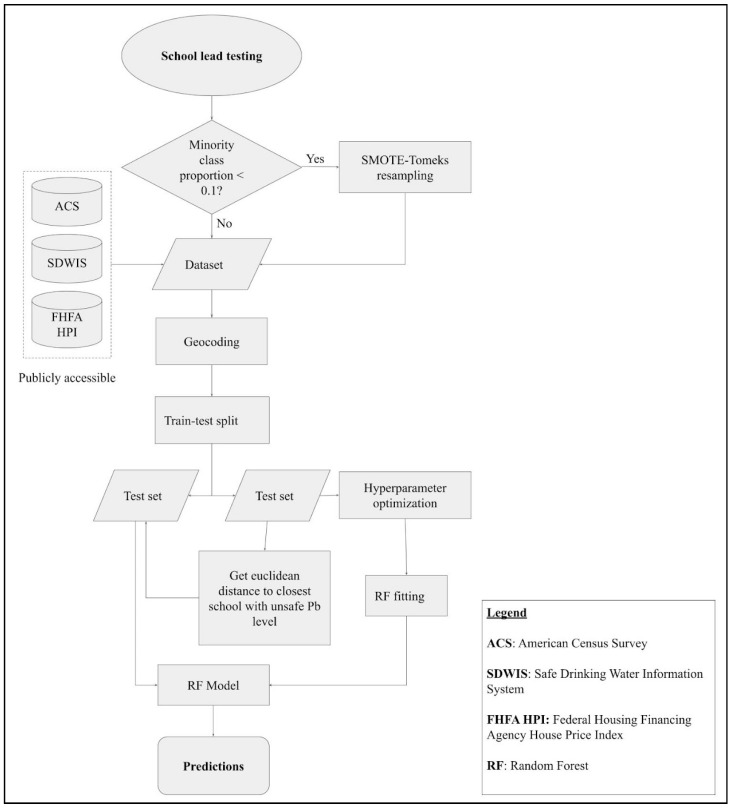
Data pipeline schematic.

The school datasets chosen were from California (*n* = 4509), Massachusetts (*n* = 1161), New Hampshire (*n* = 102), and New York (*n* = 4607). The approach described in Lobo et al. 2022 for California and Massachusetts [5] was used to create a data pipeline, schematically represented in Figure 1.

In this data pipeline, the publicly available data from testing of schools’ drinking water for lead levels (presented in Section 2.1) is collected and the class distribution is evaluated. Additional demographic, socio-economic, and water quality-related data corresponding to the zip code of each sample is added to the lead data (as described in Section 2.2). If the dataset is imbalanced, i.e., the minority-class data forms less than 10% of all data, SMOTE-Tomek Links resampling is performed to balance the class labels (detailed in Section 2.3). Based on the level of imbalance, either the resampled data or the original dataset is used for further processing. The dataset is then divided into training and test sets. The geographic Euclidean distance to the closest school in the training set with unsafe WLLs is added as an additional feature in the test set, based on results analyzed in a prior study [5]. The random forest model is tested using *K*-fold cross validation for different split sizes (as described in Section 2.4), and evaluated using metrics defined in Section 2.5.

These datasets were chosen for ease of access, and to study the effects of the size and sample variations in the dataset composition. To our knowledge, the current approach was applied to public datasets in the states of New Hampshire and New York for the first time.

A further description of the various parts of the data pipeline follows.

### 2.1. School-Level Lead Datasets

For this study, a positive value in the model was defined as a school with at least one on-site water source with a lead concentration measured as 15 ppb or higher. The work in this area has historically utilized three distinct threshold levels for lead concentration based on the allowable lead content in bottled drinking water (5 ppb), the World Health Organization regulations (10 ppb), and the action levels specified in the federal guidance for public water suppliers (15 ppb). In the majority of the US states, mitigation has been required when lead is present in water at 15 ppb or higher and publicly available datasets have published results in keeping with this threshold [7]. As described below, there are additional protocol differences regarding the collection of water samples for measuring lead. In this study, we ignore the difference within the protocols, as no correction process has been published to convert the lead values from one protocol to another.

The sampling protocols require that the water samples should be collected from water that has been stagnant within the pipes for a specified period. This duration, known as the “stagnation period”, and the volume of the water sample collected, vary from state to state. The stagnation period and sampling quantity required for “first draw” sampling varied between states as follows. New Hampshire and California required 1 L of water obtained after a 6 h or longer stagnation period [8,9]. Massachusetts required 250 mL bottles and an 8 h or longer stagnation time [10]. New York recommended that public schools and schools under the Boards of Cooperative Educational Services use 250 mL bottles, and water stagnant for a minimum of 8 h and a maximum of 18 h [11]. The EPA has since 2018, in the 3Ts for Reducing Lead in Drinking Water in Schools and Child Care Facilities, recommended the use of small samples of approximately 250 mL after a stagnation period of 8 to 18 h [12].

### 2.2. Additional Socioeconomic Predictors

Socioeconomic data was aggregated on a zip code level and linked to all schools within that zip code. For brevity, we refer to our prior study [5] regarding the explanation of this approach. This data were sourced from the 5-year American Census Survey (ACS) data from 2015 to 2019, Federal Housing Financing Agency House Price Index (FHFA HPI) datasets for 2019, and Safe Drinking Water Information System (SDWIS) Federal Reporting Services. A description of the tables used to provide raw data is included in the Supplementary Materials (Table S1). The relevant tables from these databases were grouped into 4 clusters:

(1) Poverty and income: the median income and employment information and measures of income inequality among different social groups.
(2) Race-related factors: the proportion of the total population identified in different racial categories.
(3) Household-level determinants: the house prices as well as linguistic and ethnic identity
(4) Water quality data reported by the closest water utility, including source, number of people served, and all documented violations reported under the Safe Drinking Water Act.

For datasets that did not have geolocations, the ArcGIS open-source geocoding API was used to identify geographic coordinates for each point. These coordinates were used to create a feature matrix with the Euclidean distance between every pair of schools in each state. To improve the prediction method by accounting for this phenomenon, the nearest neighbor school in the training set with unsafe WLL(s) at one or more water outlet(s) was used as an additional training feature and recalculated for each training set. This was included in keeping with the analysis done by Lobo et al. 2022 [5], which demonstrated the high prevalence of lead leaching in clusters of schools situated within close geographic proximity, possibly due to similarities in demography, the time at which water infrastructure was built, or water quality.

### 2.3. Data Preprocessing

In California, bill AB 746 mandated testing of all schools for lead in drinking water by July 1, 2019. Its passage has resulted in higher levels of testing and compliance to the EPA standards for lead in drinking water [13]. This corresponded to a highly imbalanced dataset, with positive values (i.e., schools with high WLLs) forming a small minority of all samples. To prevent overfitting the model and thereby favoring predictions of the majority class (comprising schools with low WLLs), the Synthetic Minority Oversampling Technique (SMOTE)-Tomek Links sampling was deployed to generate a balanced dataset. In SMOTE-Tomek Links resampling, SMOTE oversampling is first used to generate plausible synthetic examples of the minority class label, i.e., schools with high WLLs. This is done by calculating the Euclidean distances between examples of this label and extrapolating them in the feature space to define a new example. SMOTE is followed by Tomek Links undersampling, where samples in the dataset that form pairs of nearest neighbors by the Euclidean distance but have different class labels are identified. The sample with the majority class label (low WLL) is then removed from the dataset [14]. The resampling process ensures that a balanced dataset is achieved while maintaining a clear decision boundary for classification with minimal noise.

### 2.4. Modeling

The size splits for the training and testing data varied from utilizing 80% to 10% of the dataset for training the random forest model. Random forest modeling was selected due to its ease of use and time efficiency, as well as its demonstrated out-performance of other modeling techniques, including decision trees, *k*-nearest neighbor, supported vector machine, naïve bayes and logistic regression when applied to this specific problem, as was documented and published earlier [5]. Iterative imputation was used to estimate the missing parameters in the dataset. In this method of imputation, the missing features are expressed as a function of the existing features and estimated over repeated iterations [15]. An indicator for the missing parameters was added to reduce the possibility of biases in the causal effect estimation and to account for any correlations between the missing or unreported data and unsafe levels of lead [16]. For each split size, *K*-fold cross validation using 10 folds was used to optimize the model hyperparameters. For *K*-fold cross validation, the training data was split into k unique parts. A model with a specific set of hyperparameters was trained on *k* − 1 splits and was validated on the excluded set. The process was repeated for each fold, and the best model was selected. The full code and datasets can be found in the Data Statement.

### 2.5. Model Evaluation

The hyperparameter optimization was conducted using two different metrics: (1) the precision-recall area under the curve (PR AUC), and (2) the receiver operating characteristic area under the curve (ROC AUC). These were selected in consideration of the label imbalance in all the datasets. By taking into account both precision and recall, ROC AUC and PR AUC allow for the greater penalization of the positive labels that were incorrectly classified. The hyperparameter tuning using either of the two metrics was found to produce no improvement in the model predictions, and was hence excluded from the pipeline processing. A default random forest model with an n_estimators parameter value of 500, consistent with findings in Lobo et al. 2022 [5], was utilized for all 4 states. This exclusion had the advantage of significantly reducing the processing time and computing power needed to run the model. The model was classified as acceptable if an ROC AUC score was greater than 0.7 and a PR AUC score was greater than 0.7 [17].

The mean Gini importance of the features in each feature category, as well as five features in each category with the highest Gini scores, were plotted across split sizes to identify the trends in feature importance. The Gini importance is a measure used in implicit feature selection of random forest modeling. The metric scores each feature based on the number of times it was selected at a node in the model for obtaining a split and the corresponding decrease in the entropy at that node [18].

#### 2.5.1. ROC AUC

The ROC curve plots the performance of a model at all classification thresholds; each point on the curve represents the true positive fraction on the Y-axis, and the false positive fraction on the X-axis, for a given classification threshold. The area under this curve is defined as the AUC score.

#### 2.5.2. PR AUC

For computing the PR AUC, the precision is plotted against recall, and the area under the curve is computed. An AUC score of 0.5 is typical of a model that randomly assigns positive and negative values, whereas an AUC score of 1 indicates a perfect model. The individual precision and recall scores of the split sizes in each state are included in the Supplementary Materials (Figure S1).

## 3. Results

**Figure 2:**
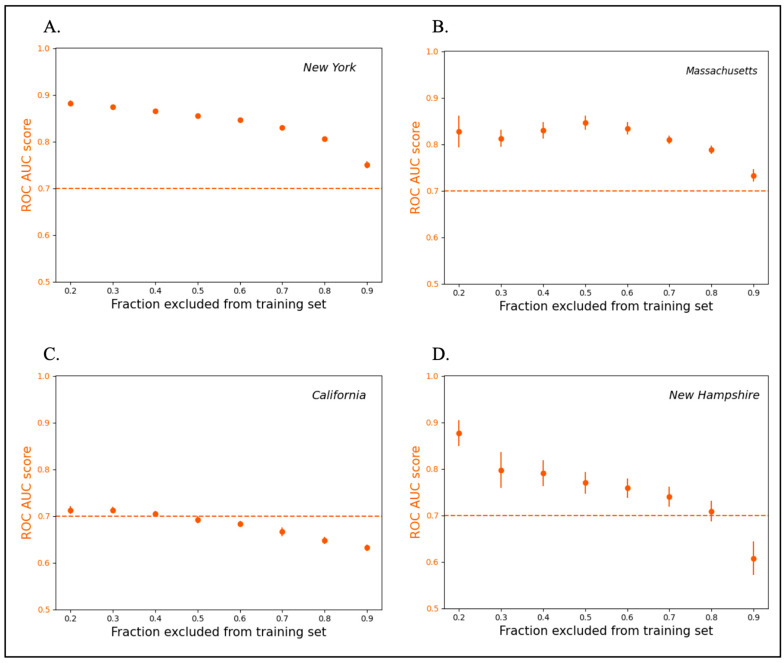
ROC AUC scores with decreasing proportion of schools used in training the model. The dashed horizontal line at ROC AUC score of 0.7 reflects what is considered acceptably good value for ROC AUC. However, any value better than 0.5 reflects a performance better than a random guess. (**A**,**B**,**D**) achieve ROC AUC scores above the 0.7 cut-off as long as 10% of schools are used to train the model. In (**C**) (California), a minimum of 50% of schools are needed to achieve an acceptable ROC AUC score, but model utilization still confers an advantage over random selection (ROC AUC = 0.5) for all split sizes.

**Figure 3:**
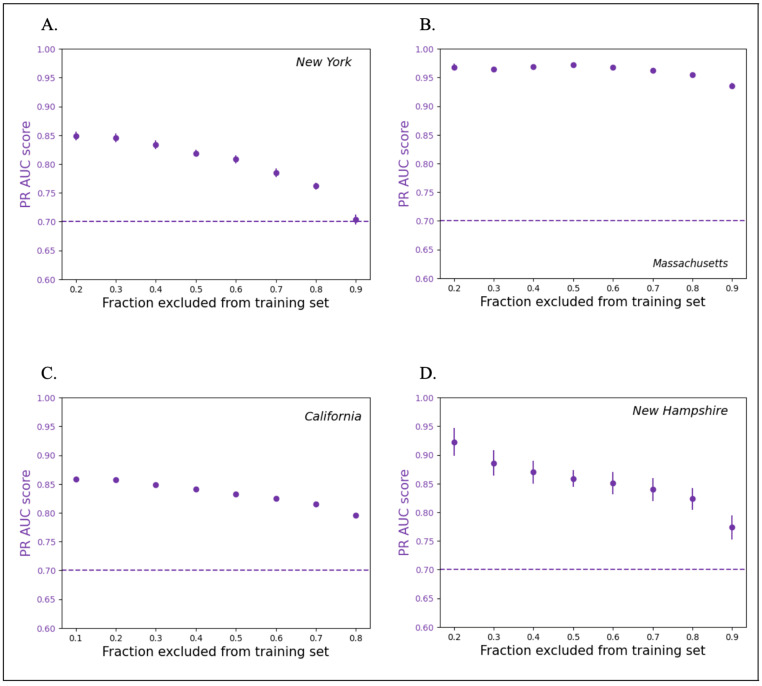
PR AUC scores with decreasing proportion of schools used in training the model. Using this metric, the models perform above the cut-off of 0.7 for all four states, despite the variability in proportions of high WLL schools. In (**A**,**B**,**D**), high WLL schools make up a relatively high fraction of all schools (i.e., more than 20%). In (**C**) (California), these schools make up a smaller fraction (about 6%) of the total schools.

Across the four states, the model evaluation using the ROC AUC and PR AUC metrics confirmed a successive increase in the cross-validation score with the addition of schools to the training set as predicted in Lobo et al. 2022 [5]. The models performed within the acceptable ranges defined in Section 2.5 for all split sizes across all four datasets. A safe standard for the state-level lead predictions would be to randomly test 50% of the schools to inform the model. However, our results demonstrate that testing as few as 10% of the schools (assuming an unbiased 10% sample) for use as a model training set is sufficient to enable predictions that are better than a random selection (Figure 2 and Figure 3).

The model performed best when the state dataset was balanced and did not require resampling. In the case of California, the sites with drinking water outlets that had positive rates (i.e., unsafe levels of lead) comprised 5.7% of the dataset, in contrast to New York (Figure 2A and Figure 3A) and Massachusetts (Figure 2B and Figure 3B), which had positive rates of 33.6% and 58.4% respectively. As a result, the model trained on the California data required larger training sets (more than 50% of the total schools) for the performance metrics to reach values considered good (Figure 2C and Figure 3C), consistent with the imbalance in the California dataset. Nevertheless, a clear threshold could be identified for the minimum number of schools needed to train the model. Examining the four states, the decline in the model accuracy with the training datasets containing 50% or fewer schools in a state indicates that this approach can require an initial random sampling of as much as 50% of the schools to be used as the model inputs. The inherent variabilities in the sampling protocol in publicly available lead data for each state are likely to have influenced the dataset balance. However, as in the case of New Hampshire (Figure 2D and Figure 3D), not adhering to the EPA best practice in lead sampling was not found to have necessarily influenced the model performance. Additionally, in New Hampshire, despite the anomalously small dataset size, favorable results were obtained with respect to both scoring metrics.

Across datasets, the two groupings of the census features, “poverty_and_income” and “household_social_determinants”, had the greatest cumulative importance. The low importance of “utilities_data” is consistent with the prevalence of lead contamination close to the point-of-use due to the corrosion of the lead service lines, pipes, fixtures, and solder [19]. The outlier state to this trend was New Hampshire, where the distance to the nearest WLL-positive school had the highest importance over all the train-test splits. The anomalous results for New Hampshire are likely a result of a smaller population (1.3 M) in comparison to the other states surveyed, resulting in a smaller overall sample size for the model. New Hampshire published findings for 102 schools, which is significantly smaller than the records for New York (*n* = 4607), Massachusetts (*n* = 1161), and California (*n* = 4509). The findings for each individual training set size remained consistent with the findings in Lobo et al. 2022 [5], with the distance to the nearest positive school being the most important feature in New York and New Hampshire as well as California and Massachusetts. As 72% of the schools in NH had unsafe WLLs, it is likely that the proximity was a stronger predictor of other schools with unsafe WLLs owing to the low population density and geographic clustering.

**Figure 4:**
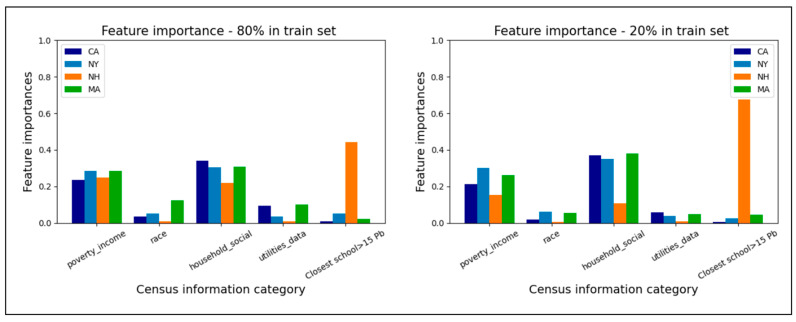
Normalized feature importance with decreasing train set sizes—cumulative Gini scores for all included features.

After answering the main practical questions regarding the size of the training dataset, we also explored a secondary question: how the importance of the different features varies with the different amounts of training data. With progressively smaller training sets, it was observed that the normalized importance of the socioeconomic features increased, and the model placed less emphasis on the smallest Euclidean distance to the nearest positive school in the training set (Figure 4). To determine the extent to which individual features impact the model, the Gini score average of the most important five features in each feature category was also plotted and are included in the Supplementary Materials (Figure S2). The findings from all states show that there were no singular features with a comparatively high Gini importance, which is to be expected given the correlation between the different features from the same census tables. The feature selection did not offer any significant advantage in terms of model speed or accuracy.

The training and *K*-fold cross-validation of the model on all the available features was found to be efficient with respect to the time efficiency and processing power. The feature selection does not offer a significant advantage in terms of the speed or performance. The details of the system used to run these models are detailed in the Supplementary Materials to support the viability of the modeling process (Section S1).

## 4. Discussion

It is imperative to note that the approach described in this study is not intended to suggest a causal relationship between the socioeconomic indicators and lead exposure through drinking water. These results are reflective of previous studies that emphasize the disproportionate prevalence of lead service lines and fixtures in communities of color and low-income areas [20]. Hence, we expect the modeling accuracy to deteriorate if progress is made towards the LCRR’s commitment to prioritizing the lead reduction measures in disadvantaged communities. This approach is also in no way a substitute for comprehensive state-wide lead testing in schools, given the ethical implications of the imperfect and inaccurate model predictions. Rather, the use of random forest modeling can significantly improve the efficiency and accelerate the positive impact of lead testing by enabling the informed prioritization of vulnerable locations. As the model works best on balanced datasets, this pipeline process can be utilized for the initial diagnostic assessments in states where testing is in its early stages to inform the testing protocols. With more imbalanced datasets, resampling techniques such as SMOTE-Tomek Links are required for effective modeling.

This approach is limited by the availability and accuracy of the publicly available lead testing data. Although a training set comprising between 10% and 50% of the schools was sufficient for the modeling in these four states, the process was dependent on using randomly selected data for training. The model is hence at risk of being biased in a context where schools in socioeconomically advantaged areas are more likely to have resources for lead testing. The differences in the school sampling protocol, imprecise water quality data from water utilities, lack of relevant socioeconomic data, etc. presented challenges to the modeling efficacy. Furthermore, while all the features were aggregated and linked to each school based on the zip code, the geographic discrepancies between the zip code regions and school districts are likely to have affected the model performance in some cases.

Our results suggest that random forest modeling has the potential to assist decision-makers in conducting state-wide lead testing in schools. By using this approach to prioritize the provision of technical, managerial, and financial support to at-risk communities, random forest modeling could be used to build a bottom-up testing strategy where the data from new sites are continuously added to the training set and used to continuously refine the model. Additionally, modeling may be used to inform existing citizen science that aims to monitor lead levels using crowdsourced data [21]. The augmentation of the relevant poverty, income, and household-specific data with citizen testing can help account for the variabilities in the testing methodology that are not captured by the state testing protocols. While this technique potentially presents the challenges of additional human error, citizen science approaches to lead testing in childcare facilities have proven to be beneficial within a framework that provides adequate training in the best practices for water sampling [22].

## 5. Conclusions

The LCRR emphasizes monitoring the water lead levels in all schools and child-care facilities; however, the financial and personnel resources required to conduct such exhaustive monitoring within the proposed time frame are limited. In this context, our results suggest that random forest modeling can be a useful approach to prioritize schools for testing, based on the predicted increased risk of high levels of lead in water. To deploy these models for statewide testing, it is important to test a randomly selected small fraction of the schools in each state, a fraction that varied between 10% and 50% in the four states studied here, to form a training set for further model-based recommendations on where to test. Although the scope of this study was limited to four states, the success of the random forest modeling, despite the inherent variations in these datasets, suggest the transferability of this process to other states within the United States, as well as internationally. Further analysis will be needed to study the impact of the different socioeconomic and water quality feature sets available in different geographic areas on the efficacy of this modeling approach.

The predictions obtained through the random forest modeling reaffirm the disproportionate impacts of elevated WLLs on schools serving primarily low-income and marginalized populations and the need to prioritize testing at these locations. By solely utilizing open-source machine learning packages and datasets, this study aims to enable and support community-centric testing strategies.

## Data Availability

All code can be accessed in our Github repository at https://github.com/gadgil-group/School_Pb_water/ (accessed on 24 May 2023).

## Supplementary Materials

The following supporting information can be downloaded at: https://www.mdpi.com/article/10.3390/ijerph20196895/s1, Section S1: Model memory and time specifications; Table S1: Census tables used to provide socioeconomic and demographic data; Figure S1: Precision and recall scores for models at each split size; Figure S2: Normalized feature importance with decreasing train set sizes - Average of 5 features in each feature category with highest Gini score.
