# Supplementary material for: Data-Sparse Prediction of High-Risk Schools for Lead Contamination in Drinking Water: Examples from Four U.S. States
Source: Int J Environ Res Public Health. 2023 Oct 8;20(19):6895. doi: 10.3390/ijerph20196895 (PMC10572968; doi:10.3390/ijerph20196895)
Supplement: Supplementary file 1 [file ijerph-20-06895-s001.zip › ijerph-2556569-supplementary.pdf]

Section S1: Model Memory and Time Specifications

The model was observed to have an average run-time of approximately 770 s on a 2020 Apple MacBook Air laptop with a 3.2 GHz M1 processor and 8 GB RAM capacity.

Table S1. Census tables used to provide socioeconomic and demographic data.

Table Identifier	Description
B17020	Poverty By Age
B29003	Voting Age By Poverty
B29004	Median Household Income In The Past 12 Months (In 2019 Inflation-Adjusted Dollars)
B29002	Voting-Age Population By Educational Attainment
B19101	Family Income In The Past 12 Months (In 2019 Inflation-Adjusted Dollars)
B19083	Gini Index
B21004	Median Income In The Past 12 Months (In 2019 Inflation-Adjusted Dollars) By Veteran Status By Sex For The Civilian Population 18 Years And Over With Income
B17005	Poverty Status In The Past 12 Months Of Individuals By Sex By Employment Status
B02001	Race
B16009	Poverty Status In The Past 12 Months By Age By Language Spoken At Home For The Population 5 Years And Over
B17019	Poverty Status In The Past 12 Months Of Families By Household Type By Tenure
B17021	Poverty Status Of Individuals In The Past 12 Months By Living Arrangement
B23001	Sex By Age By Employment Status For The Population 16 Years And Over

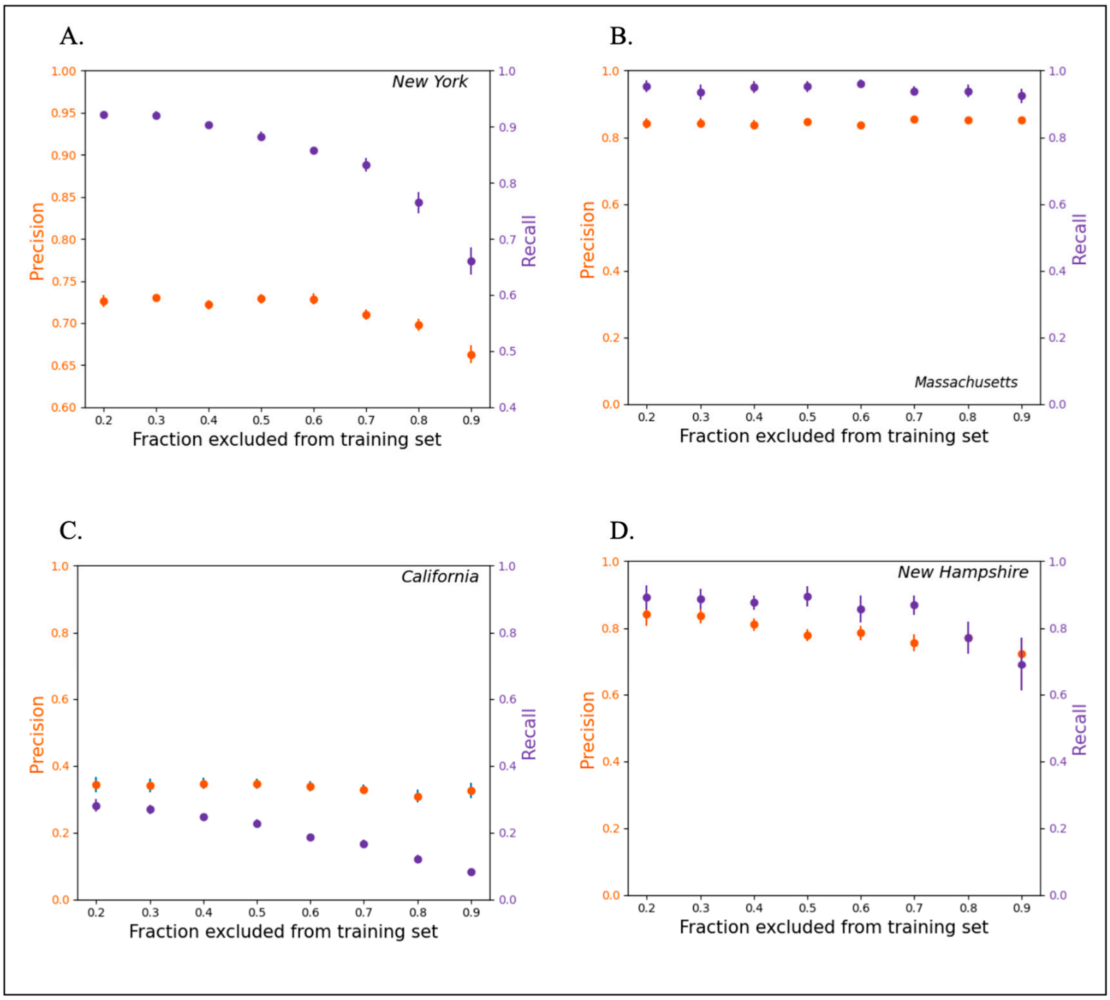

Figure S1. Precision and recall scores for models at each split size.

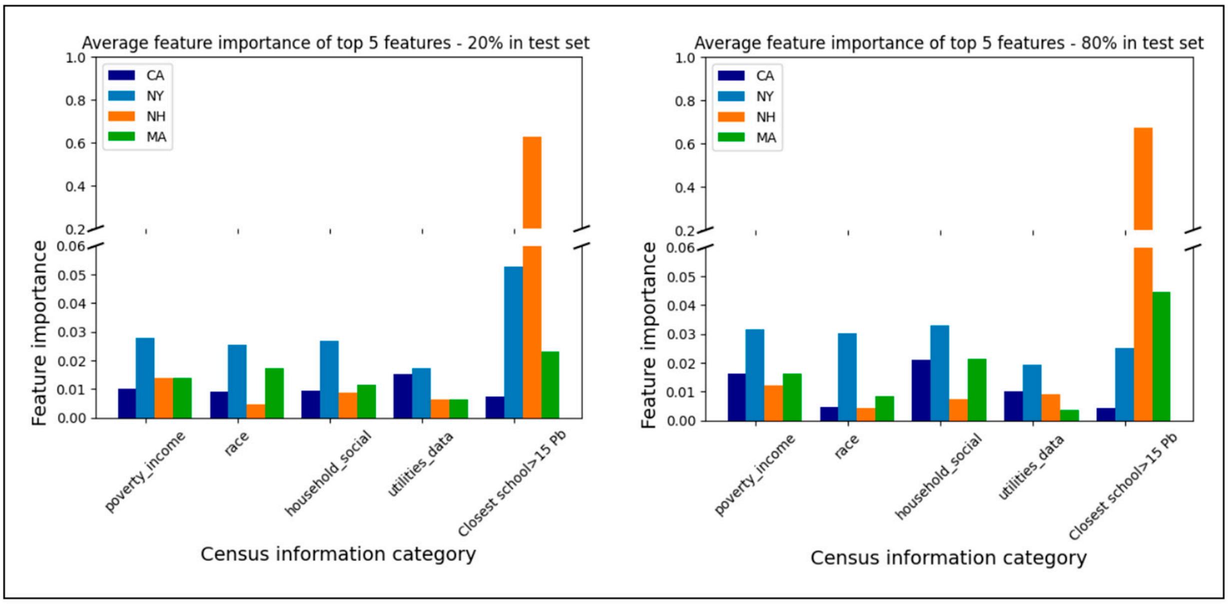

Figure S2. Normalized feature importance with decreasing train set sizes—Average of 5 features in each feature category with highest Gini score.
